# Relationship between peripheral vasospasm and visual field progression rates in patients with normal-tension glaucoma with low-teen intraocular pressure

**DOI:** 10.1371/journal.pone.0250085

**Published:** 2021-04-15

**Authors:** Do Young Park, Jong Chul Han, Eun Jung Lee, Changwon Kee

**Affiliations:** 1 Department of Ophthalmology, Yeungnam University College of Medicine, Yeungnam University Hospital, Daegu, Korea; 2 Department of Ophthalmology, Sungkyunkwan University School of Medicine, Samsung Medical Center, Seoul, Korea; National Eye Institute, UNITED STATES

## Abstract

**Purpose:**

To investigate the association between peripheral vasospasm and the visual field (VF) progression rate in patients with normal-tension glaucoma (NTG) with low-teen intraocular pressure (IOP).

**Methods:**

The finger temperature of 113 NTG patients was measured before and after exposure to ice water using a Temperature gun (cold pressor test). These patients had confirmed VF progression, despite a low-teen IOP during a follow-up period of >5 years. VF progression rates were calculated as the slope of the visual field index (VFI) and mean deviation (MD) over time. Demographic, systemic, and ocular factors and VF progression rates were compared, based on the cold pressor test results. A regression analysis was used to investigate the factors affecting VF progression rates.

**Results:**

Mean age, initial IOP, mean IOP during the follow-up period, and initial VF MD were 57.1 years, 15.8 mmHg, 12.0 mmHg, and -5.2 dB, respectively. When patients were divided into two groups (less vasospasm and more vasospasm) according to changes in temperature after exposure to ice water, the VF progression rate was significantly faster in the group with more vasospasm. In a multiple regression analysis, older age, worse initial VF MD, and greater decrease in finger temperature after ice water exposure were significantly associated with faster VF progression rates.

**Conclusion:**

An excessive drop in finger temperature after exposure to ice water was significantly associated with faster VF progression in patients with low-teen NTG. This suggests that the blood flow in the optic nerve head may also be disturbed by peripheral vasospasm, accelerating glaucomatous damage regardless of IOP.

## Introduction

It is well known that several vascular factors besides intraocular pressure (IOP) are associated with the development and progression of glaucoma, especially in those with normal-tension glaucoma (NTG) [1–3]. Low systemic blood pressure (BP), nocturnal dip in diastolic BP, and diurnal fluctuation of BP have been widely reported to increase the risks of NTG development and progression [3–6]. As a mechanism, ischemic damage from the low ocular perfusion pressure and ischemic reperfusion have been suggested to induce axonal damage through reactive oxygen species [2, 7, 8]. Repetitive tissue damage from ischemic reperfusion injuries are observed in various vasospastic disorders, including Raynaud disease, migraine, and variant angina, which show over-reactivity to common stimuli, such as cold, stress, or hypoxia [9]. Similarly, vasospasm has long been an issue for its role in the pathogenesis of glaucoma, especially NTG, in terms of development and progression [10, 11]. We hypothesized that vasospasm, which could interfere with ocular perfusion and cause ischemic reperfusion injury, could cause an additional harmful effect on the optic nerve head (ONH) where glaucomatous damage has already occurred.

One problem is that the ability to measure ocular blood flow is necessary to evaluate vasospasm within the ONH. Considering that ocular blood flow is important for the pathogenesis of NTG, various imaging modalities have been introduced to measure ocular blood flow, including Color Doppler imaging, Laser Doppler Velocimetry, Laser Speckle flowgraphy, and optical coherence tomography angiography [2]. However, these methods are currently not widely used to assess ocular blood flow, because they are not standardized, the results are observer-dependent, and their reliability has not been confirmed [12]. Therefore, indirect methods have been used to estimate the ocular blood flow from other peripheral vessels, such as patients’ complaints about cold hand/feet, nailfold capillaroscopy, or laser Doppler flowmetry of the hand/feet. Previously, abnormal findings of nailfold capillaroscopy and laser Doppler flowmetry have been reported in NTG patients [13–15].

The cold pressor test is used to evaluate the cardiovascular response that occur when an individual’s hand is immersed in ice water. It has also been used to evaluate peripheral vasospasm in patients with non-ocular disease, such as Raynaud’s phenomenon, other rheumatic diseases, and peripheral arterial obstructive disease by measuring ice-water-induced changes in temperature or blood flow in hands or fingers assessed by various modalities, such as thermography or laser Doppler flowmetry [16–19]. To the best of our knowledge, no studies have measured the finger temperature pre- and post- cold exposure, which can provide objective and quantitative data for peripheral vasospastic response in patients with NTG. Moreover, no studies have evaluated the association between peripheral vasospasm and the rate of the VF progression in NTG patients, particularly in those who have maintained low-teen IOP.

We aimed to assess whether the progression rate of VF in patients with low-teen NTG was associated with abnormal vasospastic response, measured by ice-induced temperature changes in the fingers.

## Materials and methods

### Subjects

Patients diagnosed with and treated for NTG were consecutively enrolled according to the following inclusion criteria: 1) age at diagnosis >40 years; 2) follow-up (FU) for >5 years; 3) more than one reliable VF test and disc photograph per year; 4) mean IOP during FU <13 mmHg or showing a reduction >30% from baseline; 5) progression of glaucoma was confirmed in at least one eye when the patients visited the glaucoma clinic in Samsung Medical Center between January 2017 and December 2019; and 6) the cold pressor test was performed during the same period. The study was approved by the Samsung Medical Center Institutional Review Board (IRB) and followed the tenets of the Declaration of Helsinki. Based on the nature of the retrospective study, the requirement to obtain informed consent was waived by the IRB of the Samsung Medical Center.

NTG was defined by the following criteria: 1) the presence of typical glaucomatous optic disc changes, including increased cupping and focal or diffuse loss of neuroretinal rim at diagnosis; 2) glaucomatous VF defect in at least two consecutive tests; 3) open-angle on gonioscopic examination; and 4) IOP ≤21 mmHg at each of the two visits without the use of an antiglaucomatous agent. A glaucomatous VF was defined when at least two of the following three criteria were satisfied: 1) a cluster of three points with probability <5% on the pattern deviation map in at least one hemifield, including at least one point with a probability <1% or a cluster of two points with a probability <1%; 2) a glaucoma hemifield test result outside the normal limits; or 3) a pattern standard deviation of 95% outside the normal limits [20]. Patients with any one of the following conditions were excluded: 1) eyes with MD less than -20 dB at baseline; 2) ocular surgeries excluding cataract extraction during FU; 3) history of ocular trauma, uveitis, angle-closure, pigmentary, or exfoliation glaucoma; or 4) any other ocular or systemic disease that could have caused optic nerve head and/or VF changes. To minimize the effects of IOP fluctuation, IOP was measured once in the morning and once in the afternoon.

At the first visit, a comprehensive ophthalmic examination was performed on all patients, including slit-lamp biomicroscopic examination, Goldmann applanation tonometry, gonioscopic examination, dilated stereoscopic examination of the disc, color and red-free fundus photography (TRC-50DX model; Topcon Medical System, Inc., Oakland, New Jersey, USA), automated perimetry with the Swedish interactive threshold algorithm standard using a central 30–2 Humphrey field analyzer (HFA model 640; Humphrey Instruments, Inc., San Leandro, California, USA), manifest refraction, axial length measurement (IOLMaster; Carl Zeiss Meditec, Jena, Germany), and ultrasonographic pachymetry (Tomey SP-3000; Tomey Ltd., Nagoya, Japan). Initial baseline IOP was determined as an average of ≥2 measurements without using IOP-lowering medications. IOP was measured every time patients visited the clinic, and mean IOP was represented as the average of all values obtained during the FU period. The VF test was performed, and disc/retinal nerve fiber layer (RNFL) photographs were taken at 6–12-month intervals.

Glaucoma progression was assessed based on structural changes in the optic disc and/or RNFL and corresponding functional changes on VF tests. We determined the endpoint of the FU as the time when progressive VF was confirmed together with optic disc/RNFL changes. Structural progression was considered if the following changes were confirmed: changes in neuroretinal rim thinning or notching, changes in the vascular configuration of the optic disc, and increase in the depths or widths of RNFL defects or new RNFL defects. VF progression was determined by event-based analysis using the Guided Progression Analysis (GPA) software from the Humphrey Field Analyzer. It was defined as a significant decrease from the baseline (initial two reliable VF test) pattern deviation at three or more of the same test points on two or three consecutive VF tests, in which the software classified cases as “possible progression” or “likely progression,” respectively. Two experienced glaucoma specialists (DYP, JCH) assessed optic disc and RNFL changes and VF progression; both were blinded to the subjects’ identity and all other test results. Discrepancies were resolved through discussion, and if needed, CK was consulted. If glaucomatous progression was confirmed in both eyes, the eye showing worse MD was primarily used for analysis; the other eye was considered the fellow eye. The VF progression rate was calculated for each eye using linear regression analysis of the visual field index (VFI) and MD values over time, wherein the progression rate is the slope expressed in %/year and dB/year, respectively [21].

### Cold pressor test

The patients were brought into the room 10 minutes before the test initiation to get them adjusted to the room temperature (20–23°C). The patients were not allowed to use nicotine or caffeine on the day of the exam. If the patients had a fever, the exam was rescheduled and conducted after the patient recovered. If patients were taking Ginko Biloba, they were instructed to stop taking it at least 2 weeks before the examination. This test was performed using the MultiLab Series II (Unetixs Vascular Inc. US). The patients’ hands were immersed in ice water for 60 seconds and then monitored for the following 5 minutes. The temperature of ice water was monitored and maintained at 0 to 4°C during the test. Baseline temperature of the fingers was measured using the temperature gun at room temperature, and the temperature was recorded at 1, 3, and 5 minutes after removing the hand from ice water. Temperature was measured in all fingers, but measurements from the index finger were used for analysis considering the high agreement between the temperature measurement values of the fingers. BP was measured in the arm at baseline. To maintain the same conditions in all tests, a trained technician conducted the tests using the same protocol. The cold pressor test was performed in patients with confirmed progressive NTG in at least one eye despite well-controlled low-teen IOP. The cold pressor test was also conducted on normal healthy subjects who agreed to participate voluntarily.

### Subgroup analysis

For comparing the patients with less vasospasm and more vasospasm, the patients were divided into two subgroups according to the median value of % reduction in finger temperature measured 1 minute after ice water exposure (32.9%). Similarly, patients were divided into two subgroups according to the median VF progression rate (-1.3%) assessed using VFI to compare patients according to the VF progression rate.

### Statistical analyses

Clinical characteristics were compared between the two groups using an independent t-test or Mann-Whitney U test for continuous variables and the chi-square test or Fisher’s exact test for categorical variables. Characteristics were compared among three groups using ANOVA, and the Turkey’s HSD procedure was used for multiple comparisons. A P-value of <0.05 was considered statistically significant. The linear regression model was employed to calculate the VF progression rate, which is the slope of the MD or VFI. To identify the factors associated with the VF progression rate, univariate and multivariate regression analyses using a generalized linear model were performed. IOP values were corrected for CCT and used in the statistical analysis. Characteristics with a P-value <0.1 in the univariate analysis were included in the multivariate analysis. Statistical analyses were performed with SPSS software version 24.0 (SPSS, Inc, Chicago, Illinois, USA) and R statistical packages version 3.5.3 (http://www.R-project.org).

## Results

From January 2017 to December 2019, 116 Korean patients were identified to have progressive NTG despite adequate IOP control, satisfying the inclusion criteria. Three patients were excluded who did not undergo cold pressor test owing to poor general condition; finally, 113 patients were included. Among the 113 patients, the IOP of 9 patients with relatively rapid VF progression or with severe VF defect at a young age were monitored, at two-hour intervals for 48 hours to rule out the possibility of IOP spikes throughout the day. None of these patients had an IOP of 21 mmHg or higher during the 48 hours of IOP monitoring.

Baseline characteristics of the patients are described in Table 1. Mean age of the patients was 57.1, and the mean FU duration was 7.3 years. Initial IOP and Average IOP during FU period were 15.8 ± 3.1 mmHg and 12.0 ± 1.9 mmHg (mean ± SD), respectively. Initial MD, pattern standard deviation (PSD), and VFI of the patients were -5.22 dB, 7.66 dB, and 85.2%, respectively. The VF progression rate assessed by MD and VFI were -0.46 dB/year and -1.7%/year, respectively.

**Table 1:** Clinical characteristics of the NTG patients.

	Total subjects (n = 113)
Age, years	57.1 ± 11.9
Sex ratio, male/female	42/71
Hypertension (n/y)	96/17
Diabetes mellitus (n/y)	105/8
Central corneal thickness, um	516.9 ± 59.8
Axial length, um	24.9 ± 1.54
Duration of FU, year	7.3 ±.1.9
Number of VF examinations	8.1 ± 1.7
Anti-glaucoma medication, n	1.5 ± 0.8
Initial IOP, mmHg	15.8 ± 3.1
Mean IOP, mmHg	12.0 ± 1.9
Reduction in IOP, %	20.8 ± 14.6
Initial VF MD, dB	-5.2 ± 5.5
Initial VF PSD, dB	7.67 ± 4.67
Initial VFI	85.2 ± 15.2
Progression rate, dB/yr by MD	-0.46 ± 0.60
Progression rate, %/yr by VFI	-1.7 ± 1.7
Presence of DH (n/y)	72/41
Number of DH during FU	0.71 ± 1.2
Mean BP, mmHg	112.8 ± 14.1

FU: follow-up; VF: visual field; IOP: intraocular pressure; MD: mean deviation; PSD: pattern standard deviation; VFI: visual field index; DH: disc hemorrhage; BP: blood pressure.

Data are presented as mean ± standard deviation or n (frequency).

Cold pressor test showed that the baseline finger temperature was not different between NTG patients and normal control subjects, but the percentage reduction in finger temperature at 1 minute after exposure to ice water were significantly higher in NTG patients (Table 2).

**Table 2:** Results of the cold pressor test in the study subjects.

	Normal subjects (n = 20)	NTG patients (n = 113)	P value
Age, years	54.4 ± 14.2	57.1 ± 11.9	0.37^a^
Sex ratio, male/female	9/11	42/71	0.68^b^
Hypertension (n/y)	16/4	96/17	0.78^b^
Diabetes mellitus (n/y)	18/2	105/8	0.65^b^
**Results of the cold pressor test**			
Temperature of the finger, baseline	33.0 ± 2.2	32.8 ± 2.0	0.68^a^
% reduction in the finger temperature, 1 min	27.0 ± 12.3	31.8 ± 9.1	**0.042**^a^
% reduction in the finger temperature, 3 min	19.8 ± 12.5	23.5 ± 10.7	0.17^a^
% reduction in the finger temperature, 5 min	15.1 ± 13.1	18.7 ± 11.2	0.20^a^

Data are presented as mean ± standard deviation.

Statistically significant factors are shown in bold.

^a^Independent t-test or Mann-Whitney U test.

^b^Chi-square test or Fisher’s exact test.

Baseline finger temperature of the patients was not correlated with the mean arterial blood pressure (r = 0.16, P = 0.12). Interestingly, VF progression rate calculated using VFI was significantly correlated with the percentage reduction in finger temperature at 1 minute (r = 0.29, P = 0.0016) and 3 minutes (r = 0.23, P = 0.014) after exposure to ice water but not with the baseline finger temperature (r = 0.10, P = 0.27).

Patients were divided into two groups: less vasospasm and more vasospasm, based on the percentage reduction in finger temperature after exposure to ice water (median value of 32.9%) (Table 3). The VF progression rates assessed by both MD and VFI were significantly faster in the group with more vasospasm. FU duration was longer and the number of anti-glaucoma eye-drops was higher in the group more vasospasm. Female sex was predominant in the group more vasospasm. Patient characteristics according to the VF progression rate are given in Table 4. The faster VF progression rate (≤ median value of -1.3%/year) group showed older age, higher use of anti-glaucoma eye-drops, and worse VF parameters of MD, PSD, and VFI at baseline. The % reduction in finger temperature at 1 minute post-exposure to ice water was significantly higher in the faster VF progression group (Table 4). In multiple comparison, however, the % reduction in finger temperature at 1 minute after exposure to ice water in the slower VF progression group was not different from the normal control subjects (P = 0.24).

**Table 3:** Comparison of clinical characteristics and VF progression rates according to the degree of peripheral vasospasm.

	Less vasospasm	More vasospasm	P
Age, years	57.6 ± 11.8	57.1 ± 12.1	0.82^a^
Sex ratio, male/female	27/29	15/42	**0.026**^b^
Hypertension (n/y)	50/6	46/11	0.31^b^
Diabetes mellitus (n/y)	52/4	53/4	0.99^b^
Central corneal thickness, um	518.3 ± 52.7	515.4 ± 77.6	0.80^a^
Axial length, um	24.8 ± 1.6	25.0 ± 1.5	0.33^a^
Duration of FU, year	6.9 ± 2.1	7.7 ± 1.7	**0.020**^a^
Number of VF examinations	7.9 ± 1.8	8.4 ± 1.5	0.12^a^
Anti-glaucoma medication, n	1.2 ± 0.7	1.8 ± 0.88	**9.1E-05**^a^
Initial IOP, mmHg	15.7 ± 3.2	16.0 ± 2.9	0.65^a^
Mean IOP, mmHg	12.2 ± 1.9	11.9 ± 2.0	0.40^a^
Reduction in IOP, %	18.8 ± 16.3	23.1 ± 12.1	0.11^a^
Initial VF MD, dB	-5.6 ± 5.8	-4.7 ± 5.1	0.42^a^
Initial VF PSD, dB	7.63 ± 4.63	7.69 ± 4.75	0.949^a^
Initial VFI	85.2 ± 15.6	85.1 ± 14.7	0.96^a^
Progression rate, dB/yr by MD	-0.28 ± 0.58	-0.67 ± 0.56	**0.00046**^a^
Progression rate, %/yr by VFI	-1.2 ± 1.3	-2.3 ± 1.9	**0.00062**^a^
Presence of DH (n/y)	35/21	37/20	0.94^b^
Number of DH during FU	0.78 ± 1.2	0.62 ± 1.1	0.44^a^
Mean BP, mmHg	115.0 ± 14.9	110.4 ± 12.7	0.11^a^

FU: follow-up; VF: visual field; IOP: intraocular pressure; MD: mean deviation; PSD: pattern standard deviation; VFI: visual field index; DH: disc hemorrhage; BP: blood pressure.

Data are presented as mean ± standard deviation or n (frequency).

Statistically significant factors are shown in bold.

^a^Independent t-test or Mann-Whitney U test.

^b^Chi-square test or Fisher’s exact test.

**Table 4:** Comparison of clinical characteristics and cold pressor test results among patients with slow and fast VF progression.

	Slow	Fast	P
Age, years	55.2 ± 10.6	60.0 ± 12.9	**0.034**^a^
Sex ratio, male/female	21/34	21/37	0.98^b^
Hypertension (n/y)	49/6	47/11	0.35^b^
Diabetes mellitus (n/y)	53/2	52/6	0.30^b^
Central corneal thickness, um	517.8 ± 62.0	515.7 ± 77.9	0.85^a^
Axial length, um	24.8 ± 1.4	24.9 ± 1.6	0.82^a^
Duration of FU, year	7.0 ± 2.0	7.6 ± 1.6	0.10^a^
Number of VF examinations	7.9 ± 1.7	8.4 ± 1.5	0.11^a^
Anti-glaucoma medication, n	1.1 ± 0.7	1.9 ± 0.8	**3.8E-07**^a^
Initial IOP, mmHg	15.5 ± 3.0	16.2 ± 3.1	0.24^a^
Mean IOP, mmHg	12.1 ± 1.9	12.9 ± 2.0	0.56^a^
Reduction in IOP, %	18.6 ± 15.3	23.6 ± 13.1	**0.069**^a^
Initial VF MD, dB	-3.8 ± 5.2	-7.0 ± 5.5	**0.0020**^a^
Initial VF PSD, dB	6.11 ± 4.5	9.64 ± 4.07	**3.6E-05**^a^
Initial VFI	89.9 ± 12.8	79.1 ± 15.7	**9.9E-05**^a^
Presence of DH (n/y)	42/21	33/25	0.17^b^
Number of DH during FU	0.68 ± 1.1	0.74 ± 1.1	0.81^a^
Mean BP, mmHg	112.5 ± 14.1	113.2 ± 14.2	0.79^a^
**Results of the cold pressor test**			
Temperature of the finger, baseline	32.8 ± 2.0	32.8 ± 1.8	0.99^a^
% reduction in the finger temperature, 1 min	30.2 ± 9.7	33.9 ± 7.8	**0.034**^a^
% reduction in the finger temperature, 3 min	22.7 ± 11.0	24.6 ± 10.3	0.33^a^
% reduction in the finger temperature, 5 min	18.8 ± 11.7	18.8 ± 10.5	0.96^a^

FU: follow-up; VF: visual field; IOP: intraocular pressure; MD: mean deviation; PSD: pattern standard deviation; VFI: visual field index; DH: disc hemorrhage; BP: blood pressure.

Data are presented as mean ± standard deviation or n (frequency).

Statistically significant factors are shown in bold.

^a^Independent t-test or Mann-Whitney U test.

^b^Chi-square test or Fisher’s exact test.

In multivariate regression analysis, older age, worse baseline VF MD, and greater % reduction in finger temperature at 1 minute post-exposure to ice water were significantly associated with faster VF progression (Table 5).

**Table 5:** Factors associated with faster VF progression rates.

	Univariate analysis			Multivariate analysis		
	Beta	95% CI	P	Beta	95% CI	P
Age (/1 year older)	-0.029	-0.054 to -0.0034	**0.028**	-0.031	-0.056 to -0.0056	0.018
Sex, male	0.15	-0.48 to 0.80	0.63			
Hypertension, y	-0.43	-1.3 to 0.43	0.33			
Diabetes mellitus, y	-0.95	-2.16 to 0.25	0.12			
Central corneal thickness (/1 um thicker)	0.0024	-0.0028 to 0.0076	0.36			
Axial length (/1 mm longer)	0.0022	-0.21 to 0.22	0.98			
Duration of FU (/1 year more)	-0.16	-0.32 to -0.0073	**0.042**	0.0012	-0.17 to 0.17	0.99
Number of VF examinations (/1 more)	-0.14	-0.32 to 0.043	0.13			
Initial IOP, mmHg (/1 mmHg higher)^#^	0.14	-0.16 to 0.45	0.36			
Mean IOP, mmHg (/1 mmHg higher)^#^	0.21	-0.25 to 0.68	0.36			
Reduction in IOP, % (/1% higher)^#^	0.18	-0.052 to -0.43	0.12			
Initial VF MD, dB (/1 dB lower)	-0.068	-0.12 to -0.010	**0.022**	0.077	0.021 to 0.13	**0.0081**
Initial VF PSD (/1 dB higher)	-0.10	-0.16 to -0.041	0.0016			
Initial VFI (/1% lower)	-0.03341	-0.054 to -0.012	0.0020			
Presence of DH, y	-0.3111	-0.95 to 0.33	0.34			
Number of DH during FU (/1 more)	-0.06943	-0.34 to 0.20	0.62			
Mean BP (/1 mmHg higher)	0.009056	-0.016 to 0.034	0.48			
**Results of the cold pressor test**						
Temperature of the finger, baseline (/1°C higher)	0.08917	-0.067 to 0.24	0.26			
% reduction in the finger temperature, 1 min (/1% more)	-0.054	-0.086 to -0.021	**0.0015**	-0.060	-0.094 to -0.026	**0.00088**
% reduction in the finger temperature, 3 min (/1% more)	-0.036	-0.064 to -0.0077	0.014			
% reduction in the finger temperature, 5 min (/1% more)	-0.023	-0.051 to -0.0038	0.094			

FU: follow-up; VF: visual field; IOP: intraocular pressure; MD: mean deviation; PSD: pattern standard deviation; VFI: visual field index; DH: disc hemorrhage; BP: blood pressure.

Statistically significant factors are shown in bold.

^#^IOP values were corrected for CCT values and used in the analysis.

Initial VF MD, initial VF PSD, and initial VFI were strongly associated with each other; thus, only the initial VF MD was included in multivariate analysis.

On evaluating the fellow eyes in the two groups divided according to the degree of vasospasm, the presence of NTG and detection frequency of NTG progression during the FU period were not significantly different between two groups. However, VF progression rate was significantly higher in the more vasospastic group (Table 6).

**Table 6:** Comparison of the characteristics of fellow eyes of patients according to the degree of peripheral vasospasm.

	Less vasospasm	More vasospasm	P
NTG in a fellow eye, y/n (%)	42/14 (75.0%)	39/18 (68.4%)	0.57^a^
NTG progression in a fellow eye, y/n (%)	25/17 (59.5%)	30/9 (76.9%)	0.22^a^
Progression rate in a fellow eye, dB/yr by MD	0.22 ± 0.95	-0.18 ± 0.39	**0.0049**^b^
Progression rate in a fellow eye, %/yr by VFI	-0.21 ± 0.84	-0.62 ± 1.0	**0.019**^b^

NTG: normal tension glaucoma; MD: mean deviation; PSD: pattern standard deviation

Data are presented as mean ± standard deviation or n (frequency).

Statistically significant factors are shown in bold.

^a^Chi-square test or Fisher’s exact test.

^b^Independent t-test or Mann-Whitney U test

## Discussion

The patients included in the present study were those who showed glaucoma progression even though their average IOP remained at low-teen of 12.0 mmHg for 7.3 year of FU. We examined the relationship between rapid progression and peripheral vasospasm in these low-teen NTG patients. For this, we evaluated the temperature of the fingers in response to ice water as a quantitative tool representing vasospasm in NTG patients. The VF progression rate (how fast glaucoma progresses) in NTG patients with well-controlled low-teen IOP was not related to finger temperature itself but with its degree of reduction after cold exposure. These findings suggest that the exaggerated response of peripheral vasoconstriction to a particular stimulus may boost the progression of NTG, despite low IOP.

IOP is a major risk factor for NTG development and progression [22, 23]. However, since its progression occurs in about 20% of NTG patients in the setting of well-controlled IOP, it is obvious that factors other than IOP are involved in NTG progression [24]. Disturbed ocular blood flow is a significant factor in the pathogenesis of NTG [25]. Low systemic BP, especially during the night-time, has been reported to increase the progression risk in NTG, supporting a vascular mechanism of NTG [4, 6]. It is considered that unstable blood flow due to abnormal vascular autoregulation, rather than simply reduced blood flow, can cause recurrent reperfusion injury-inducing chronic oxidative stress [25].

Vasospasm is closely related to the cluster of symptoms of vascular dysregulation, including cold hands and low BP, the so-called Flammer syndrome (frequently observed in NTG patients) [8, 9]. It suggests that dysfunction of vascular autoregulation can be an important contributing factor for NTG progression. ET-1, a potent vasoconstrictor, has been thought to play a major role in dysregulating the blood flow in such conditions [9]. Increased basal plasma ET-1 levels or excessive increases in plasma ET-1 levels after cold exposure are more common in patients with NTG [26, 27]. In this study, we assessed the relationship between ice water-induced peripheral vasospasm and long-term progression rate of VF in NTG patients with low-teen IOP. Despite the low-teen IOP, NTG patients with increased vasospastic response in fingers to cold exposure showed faster VF progression rate, regardless of age and baseline severity of glaucomatous damage. It implies that if NTG patients have excessive vasospasm in their fingers, microcirculation, including at the optic nerve head, might also be impaired by vascular dysregulation, causing additional damage to the axons by ischemic reperfusion and/or oxidative stress.

Few studies have assessed the risk factors for NTG progression in patients with well-controlled low-teen IOP. Recently, progression was reported in about 35% of the eyes of patients with low-teen NTG in more than 5 years [28]. Fluctuation of diastolic BP and diurnal IOP were suggested as systemic factors associated with progression risk in NTG patients with low-teen IOP [28]. Since fluctuations in BP and IOP can periodically disturb and restore ocular perfusion, the mechanism of optic nerve damage may be similar to that caused by vasospasm. Thus, our finding of faster progression in patients with more peripheral vasospasm might be in line with the results of the previous study. Furthermore, another study showed that peripheral vasospasm in response to cold provocation, assessed by nailfold capillaroscopy, was correlated with low BP in patients with NTG [29]. It is unlikely that peripheral vasospasm itself is directly related to fluctuation in BP or IOP, but they are often reported to be accompanied by each other in NTG patients. This suggests that there may be a common predisposing factor, such as autonomic dysfunction, regulating both these factors, contributing to NTG progression [30, 31].

Since we included patients if they showed NTG progression in at least one eye, the other eye could be normal or have stable to progressive NTG. Thus, we also checked the characteristics of the other eye using the cold pressor test. Presence of NTG and detection rate of progression in the fellow eye did not differ according to peripheral vasospasm. However, the VF progression rates in the fellow eyes were faster in patients with increased vasospasm. This finding indicates that as a systemic factor, peripheral vasospasm can have a similar effect on both eyes in terms of the deterioration of NTG. A larger number of patients may be needed to confirm the difference in the detection rate of progression.

In addition to peripheral vasospasm, older age and worse VF parameters at baseline were independently associated with the VF progression rate. This is consistent with the result of a previous study that analyzed the long-term VF progression rate after DH development in patients with an average IOP of 14.5 mmHg [32]. They reported that older age and worse MD at baseline were significantly associated with rapid VF progression [32]. In the early stage of glaucoma, because structural changes precede the changes in VF and as VF progression is slow, initial VF parameters should be considered to assess the factors associated with the long-term VF progression rate [33]. In this study, most patients were perimetric with an average initial MD of -5.2 dB, and patients with advanced VF defects at baseline were excluded. Above all, the degree of peripheral vasospasm was independently associated with the VF progression rate in multivariate regression analysis.

Interestingly, DH was not involved with the factors contributing to faster VF progression rate in this study. The presence and/or number of DH have been reported to be related to the progression of glaucoma, especially NTG [28, 34]. There could be several possible interpretations of this finding. Recently, DH has been considered to develop due to capillary damage accompanied by reactive gliosis that occurs adjacent to damaged retinal nerve fibers [35]. Therefore, its occurrence may depend on the degree of interaction between capillaries and glial cells and may be independent of the rapidity of VF progression. Moreover, since we included NTG patients with confirmed progression, DH might not be related to the overall rate of VF progression. The relatively high proportion of patients (36.8%) who presented DH within the study subjects could also have affected the results. Since this study was performed retrospectively, DH may not have been observed due to its nature of absorption.

There are several limitations to this study. First, we mainly analyzed the data of the patients with NTG who had confirmed glaucomatous VF progression without including normal healthy controls. It was because this study was not performed prospectively and it was difficult to compare and interpret the results under the same conditions. Therefore, our results should not be interpreted in terms of the risk of NTG development. The results of this study show that excessive vasospastic response may be related with rapid progression of NTG in patients with low-teen IOP, and careful consideration should be taken in determining a causal relationship. Second, the number of cold pressor test results for normal healthy individuals was small. Further studies including more healthy controls are warranted. A prospective longitudinal study on NTG patients will provide valuable information about the role of vasospasm measured elsewhere, other than in the eye, on the progression of NTG.

In conclusion, it was possible to objectively evaluate peripheral vasospasm, which may be related to ocular perfusion/reperfusion by measuring finger temperature before and after cold exposure. The excessive drop in finger temperature after cold exposure was significantly associated with the risk of faster VF progression in NTG patients with low-teen IOP, suggesting that peripheral vasospasm can deteriorate glaucomatous damage regardless of IOP. A further prospective study with a larger population, including healthy people, is needed.

## Supporting information

S1 Dataset (CSV)
